# Comparing public willingness-to-pay for different low-carbon measures: A case study of Shenzhen, China

**DOI:** 10.1371/journal.pone.0319687

**Published:** 2025-03-19

**Authors:** Haiyan Hao, Jiaying Lin, Shiyong Qiu, Li Liu, Jiahuan Dai

**Affiliations:** 1 School of Humanities and Social Science, The Chinese University of Hong Kong, Shenzhen, Shenzhen, China; 2 World Resources Institute (USA) Beijing Representative Office, Beijing, China; Zhejiang Shuren University, CHINA

## Abstract

Cities in China have made progressive strides in developing low-carbon societies and experimenting with various low-carbon measures. The successful implementation of these low-carbon measures and the subsequent maintenance of relevant amenities rely on the support of local residents. However, there is limited understanding of residents' awareness and support for the different types of low-carbon measures, which can involve different trade-offs. This research addressed this research gap by surveying residents’ willingness-to-pay for five representative low-carbon measures implemented in Shenzhen, a pioneering low-carbon city in China. Surveys were collected from 14 distinct residential areas in Shenzhen, and the analysis results revealed that Shenzhen residents were more inclined to pay for low-carbon measures that directly benefit them personally, as opposed to those serving the collective good. This trend was particularly evident among educated elites. Other notable findings include: 1) respondents aware of the different low-carbon measures in effect were more likely to pay for them; 2) male respondents, new Shenzhen residents (relocated within the last 5 years), high-income individuals, and residents in aging residential areas tended to contribute higher amounts towards low-carbon measures; 3) providing detailed information on carbon mitigation effects significantly increased both the likelihood and the amount of respondents' WTP; 4) the adoption of new-energy vehicles (NEVs) is especially controversial between NEV owners and gasoline vehicle owners. These findings hold implications, such as developing targeted policies and educational interventions, to enhance public awareness and support for low-carbon initiatives, thus fostering sustainability in rapidly growing urban centers like Shenzhen.

## Introduction

The increasing risks of climate change and global warming have raised widespread interest on reducing carbon emissions. According to the Global Carbon Budget, China’s annual per-capita carbon emissions reached approximately 8.0 tons in 2021, ranking 43rd globally, yet its total emissions of about 11.5 billion tons topped the charts worldwide [1]. In response to this pressing issue, the president announced China’s commitment to adopt robust policies and measures at the 75th Session of the United Nations General Assembly, aiming at “*peaking carbon emissions before 2030 and achieving carbon neutrality before 2060”*[2], which is also referred to as the “dual carbon target”.

Shenzhen, a metropolis in southern China, has aggressively pursued low-carbon development in response to dual carbon targets by piloting a range of low-carbon initiatives [3,4]. These measures target different urban sectors, including transportation, energy, industry, and buildings, and are implemented through diverse instruments such as legislative mandates, incentives, market mechanisms, collaboration, and voluntary efforts [5,6]. For instance, Shenzhen has enacted several local ordinances to enforce stricter energy performance requirements for new buildings and ecological environment protection[7,8]. Financial incentives were provided to companies acquiring green certification, and consumers purchasing energy-efficient products and green buildings[9,10]. Additionally, the government partners with private sectors to promote renewable energy and electric vehicles (EVs). With the market mechanism, Shenzhen implemented the “*carbon trading*” pilot program to identify key carbon emitters, monitor emissions, and facilitate the trading of carbon quotas [11,12]. In the future, the general public may also participate in the carbon trading platform, exchanging carbon credits earned through their daily low-carbon behaviors under the “carbon inclusion” project [13].

Despite extensive efforts to transition Shenzhen into a low-carbon city, little attention has been paid to the societal valuations towards the various low-carbon measures. Understanding local residents’ awareness and support for these measures is not only essential but imperative for the city’s sustainable development. On one hand, numerous low-carbon projects have been meticulously tailored to residential settings, such as integrating cutting-edge green technologies in residential buildings and installing eco-friendly amenities within neighborhoods. Successful implementation of these projects and subsequent maintenance of amenities require cooperation from local residents. On the other hand, the huge financial burden historically borne by the city government to promote low-carbon endeavors poses a formidable challenge to long-term sustainability. In this regard, mobilizing local residents to become active stakeholders and investors in low-carbon development emerges as a pivotal strategy, promising not only financial robustness but also fostering a sense of collective ownership over the city’s destiny.

Previous studies have evaluated public opinions and support for various low-carbon measures and policies in both domestic and international settings. These studies have primarily focused on eliciting public willingness to pay (WTP) and measuring respondents’ valuation of specific low-carbon strategies and policies, such as green electricity [14], EVs [15], carbon emission mitigation strategies [16,17], and green housing [18]. The findings from these studies help inform strategies promoting the public adoption of low-carbon technologies and measures. Albeit insightful, these studies are mostly reactive, investigating individual low-carbon policies and technologies that raise heated debates among the public and media [19]. However, the implementation of different low-carbon measures entails different types of trade-offs. For example, enhancing the energy efficiency of buildings may lead to increased rental or maintenance costs for residential properties, while the installation of green facilities (e.g., community parks and EV charging stations) might clash with residents’ prior uses of public spaces. Despite these complexities, existing studies rarely compare residents’ diverse perceptions towards different low-carbon initiatives. Moreover, there lacks studies specifically investigating Shenzhen, a city with numerous low-carbon measures and policies in effect and more in the development.

Therefore, it is crucial to understand Shenzhen residents’ awareness and endorsement for the diverse low-carbon measures in the city. This study addresses this research gap by eliciting residents’ WTP for five representative low-carbon measures in the city [20], including upgrading to energy-saving appliances, switching to new energy vehicles (NEVs), upgrading residential energy systems, installing public charging stations, and installing green facilities in residential areas. All of the selected measures represent potential decision-making scenarios for Shenzhen residents. With surveys distributed to 524 residents across 14 residential areas in the city, the analysis reveals the heterogeneous levels of awareness and support for different low-carbon measures across social and economic groups, along with various underlying reasons. The insights gained from this research can guide city developers in devising more effective communication strategies and policy interventions to foster the development of low-carbon cities.

## Related Studies

### Public willingness to pay on low-carbon measures

Domestic and international scholars have conducted research on public WTP for various low-carbon measures across cities and countries. The findings of those studies are summarized in Table 1. These studies centered on different low-carbon measures, with many directly asking respondents’ payments for carbon emission mitigation, quantified either using percentages (e.g., mitigating 30% GHG emissions) or units (e.g., reducing one ton of GHG emission) (Table 1). The WTP of green electricity was also studied in numerous studies, often through monthly utility bills or carbon taxes. Some researchers have investigated public WTPs for specific products (e.g., EVs), services (e.g., air traveling), initiatives (e.g., constructing carbon sinks), among other low-carbon strategies. These previous studies indicate that respondents’ WTP varies across different low-carbon measures and study sites. Studies conducted in economically developed countries and regions showed higher WTP amounts.

**Table 1:** WTP on different low-carbon measures across regions in prior studies.

Research	Region	Low-carbon measure	Method	Estimated WTP
**Domestic**				
[14]	Tianjin, China	Green electricity	OE	CNY32.63 per month
[16]	Suzhou, China	Carbon emission mitigation by 30%	Not mentioned	CNY26.20 per month
[17]	Four cities in China	Carbon emission mitigation	PS	CNY169.18 - 225.23 per year
[15]	Online community, China	Electric vehicles	DC	CNY6,833 ~ CNY 29,540 compared to mainstream gasoline vehicles
[21]	China	Carbon emission reduction	PS	Monthly household WTP: 30% reduction: $4.99 60% reduction: $8.32 85% reduction: $11.18
[22]	Jiangsu, China	Green electricity	PS	Mean value ranges from CNY7.91/month to CNY10.30/month
[23]	Beijing, China	Renewable electricity	Single-bounded DC	A mean WTP of CNY28.9 and CNY21.8 for mandatory and voluntary payment vehicles.
[24]	Online community, China	Residential open (green) spaces	PS	CNY91.75 per year
[25]	Rural community in Shandong, China	Solar roof	Double-bounded DC	CNY133.57 per month
[19]	Hangzhou, China	Battery swapping stations	Double-bounded DC	CNY400.521
**International**				
[26]	Online community, Europe	Carbon emission reduction	Single-bounded DC	Mean value of €6.30 per ton of carbon dioxide (median value is €0.30 per ton of carbon dioxide)
[27]	Germany (field experiment)	Carbon emission reduction	OE	€ 16 per ton of carbon dioxide
	Germany (survey)	Carbon emission reduction	OE	€ 200 per ton of carbon dioxide
[28]	Italian	Carbon tax	PS	The median WTP ranges from €101 to €154 for an annual fixed carbon tax, and from €0.17 to €0.30 per liter for a fuel carbon tax.
[21]	Sweden	Carbon emission reduction	PS	Monthly household WTP: 30% reduction: $21.70 60% reduction: $39.54 85% reduction: $54.24
	The U.S.	Carbon emission reduction	PS	Monthly household WTP: 30% reduction: $17.27 60% reduction: $27.95 85% reduction: $36.43
[29]	The U.S.	Carbon emission reduction	PS	The mean value ranges from $79-89 per household annually for reducing 17% GHG emissions by 2020.
[30]	The U.S.	Carbon tax	OE	$15 – 20 per month
[31]	The U.S.	National Clean Energy Standard (NCES)	Bidding	Mean value of $162/year for NCES.
[32]	Italian	Carbon emission reduction	DC	€133 per ton of carbon dioxide
	Czech	Carbon emission reduction	DC	€94 per ton of carbon dioxide
[33]	Dresden, German	Carbon Capture and Storage (CCS)/renewable power delivery	DC	A 10.85€ monthly price premium for an increase of 10% renewables in the mix and a 1.54€ monthly price premium for a 10% increase of CCS power.
[34]	Sweden	Carbon emissions from air travel	Bidding	Mean value of €55/ton of CO_2_ for climate surcharge on short-distance flights; €36/ton CO_2_ for climate surcharge on long-distance flights; €32/ton CO_2_ for climate surcharge on fuels; €14/ton CO_2_ for voluntary
[35]	Australia	Carbon farming	DC	$1.13 per month or $19.2 per year
[36]	Dutch	Low-carbon residential heating	DC	About 30% of households’ current energy bill.

Previous studies have also highlighted the impact of elicitation methods on survey results (Frew et al., 2003; Oerlemans et al., 2016). Generally, there are five types of elicitation methods: 1) *Open-ended (OE)* questions that let respondents state their WTP valuation in an unbounded and unprompted manner; 2) *Payment scale (PS)* questions that provide respondents with a prespecified and ordered list; 3) *Closed-ended (CE)* format that requires respondents to make a dichotomous choice (i.e., accept or reject) at predetermined offer value. This method can be single-bounded CE or double-bounded CE. For the single-bounded option, the interviewer only made one offer and survey respondents’ dichotomous decision. For the double-bounded option, the interviewer will make two offers and the value elicited in the second offer will be higher or lower than the elicited value in the first offer based on the response to the first offer. 4) *Bidding or bargaining* involves the interviewer making successive offers that the respondent can accept or reject, with adjustments based on previous responses; and 5) *Discrete Choice (DC)* experiments that let respondents choose from an array of provided options, e.g., EVs with different performance metrics.

Researchers have compared the WTP evaluation results for different elicitation methods. For example, Frew et al. (2003) compared the estimation results of OE, CE, and PS and found that PS can yield similar results as OE, which is lower than the CE elicitation. Oerlemans et al. (2016) studied the different sources of errors for estimating WTP values and suggested that PS or multi-bounded CE approaches can yield more reliable results. Vossler and Welsh (2003) found that multiple-bounded CE yield significantly higher results than ordinary dichotomous choice method. Other researchers have also used real-world and simulation data to examine the biases present in the single-bounded and doubled-bounded CEs (Kanninen, 1995; Calia & Strazzera, 2000). Recommendations that the bid design should focus on the range between 10 – 90% and the inclusion of a large sample size can reduce the biases were made.

### Factors influencing public WTP to low-carbon measures

Researchers studying residents’ WTP for low-carbon measures have identified various factors influencing respondents’ decisions. These factors can be categorized into three main groups, i.e., socio-demographic factors; endogenous factors, and exogenous factors, as summarized below.

### Socio-demographic factors

Many researchers have explored the relationship between respondents’ WTP and their socio-demographic characteristics. Factors, such as age, education attainment, income level, occupation type, and gender, were found to influence respondents’ WTP for low-carbon measures [14–18,21,25,37]. However, these studies may reach different conclusions regarding whether these variables pose a positive or negative impact on estimated WTP, likely due to differences in contexts (locations and time) and the types of low-carbon measures examined. Nevertheless, most scholars agree that individuals with high income and education attainment are more likely to pay for low-carbon measures and pay higher amounts for them [14–17,25,36–38]. Household-related factors, such as household size, home ownership, and marital status, also play a significant role. The general idea is that respondents who own houses, are married, and have children tend to exhibit greater environmental responsibility and are more willing to pay for low-carbon measures, such as green housing and energy-saving appliances [14,17,18]. Additionally, some studies also suggested the impact of political affiliations on respondents’ WTP on low-carbon measures. Studies in the U.S. suggest that Democrats are more supportive of low-carbon policies compared to Republicans and other groups [21,29,31]. In China, some researchers have also showed that Communist Party members exhibit a higher WTP for low-carbon measures [17].

### Endogenous factors

Research into endogenous cognitive factors yields more consistent conclusions compared to socio-demographic factors. Scholars have found that respondents who exhibit greater awareness of climate change or environmental issues [14,16,17,39], perceive personal responsibility [21,35]; possess certain low-carbon knowledge [14,37], personal values [37], and trust in government [14,16,25,40,41] are more willing to pay for low-carbon measures.

In addition to these cognitive factors, perceived additional benefits associated with low-carbon projects also influence respondents’ WTP, such as reduced utility bills from green electricity projects and energy-saving appliances [42], and increased housing values in green housing projects [18]. Additionally, some researchers have indicated that respondents’ personality traits, such as positive, susceptible, responsible, and compatible, also impact their preferences for engaging in low-carbon initiatives [25,43].

Place attachment has been specially investigated in [44], where it was measured by the personal, social, and environmental bonds shared between respondents and places. The analyses indicate that respondents’ subjective satisfaction with the living environment, including housing size, neighborhood safety, and accessibility to green spaces, correlates positively with higher WTP for low-carbon lifestyles. Similar findings were drawn in [17], which showed that respondents satisfied with their current life were more likely to pay for carbon emission reductions. Similar to place attachment, some studies suggested that residents with higher psychological ownership of the places or objects for improvement were more willing to sacrifice and, consequently, contribute to higher WTP amounts [40].

### Exogenous Factors

Relatively fewer studies investigated the influence of exogenous factors. One such factor discussed in the literature is *social norm*, which refers to how surrounding “reference group” behaves and perceives low-carbon development. Researchers showed positive impacts of social norms on WTP for low-carbon measures [18,37]. Some studies also suggested the positive influences of social interaction, such as network size and relationship strength, on low-carbon consumption behaviors [40,45,46].

Additionally, studies investigated the influences of *information* on respondents’ WTP on low-carbon measures [18,39]. In [18], the authors showed that respondents with higher education attainments were less likely to be influenced by the external information provided for low-carbon measures, exhibiting the “anchoring” effect. A field experiment conducted in [27] showed that a simple information treatment could increase average WTP for carbon mitigation from zero to €16 per ton of carbon dioxide.

Moreover, *environmental factors* can influence respondents’ WTPs on low-carbon projects. In [26], the authors found that the *temperature* at the time respondents fill out the survey could impose a positive impact on respondents’ WTP. This is plausible as respondents may be more aware of global warming if they were exposed to higher temperatures. Similarly, researchers in [38] found that respondents living in cities exposed to higher levels of air pollution were more willing to pay for clean air initiatives.

Some researchers have also examined the impacts of *payment instruments* on respondents’ WTP to low-carbon initiatives. For example, some researchers found that respondents are less willing to pay for renewable energy if the payments were collected voluntarily rather than mandatory [23]. In contrast, a study conducted in Korea showed that people are more willing to pay higher amounts with facilitative policy instruments (e.g., subsidy) compared to sanctioning policy instruments (e.g., tax) for new energy policies [47].

## Method

### Hypothesis development

The primary objective of this study is to estimate the willingness-to-pay (WTP) of Shenzhen residents for existing and emerging low-carbon measures. The Shenzhen government has implemented different low-carbon measures. Our research group selected five representative measures from a previous study, which lists feasible low-carbon measures for Shenzhen based on expert recommendations [20]. The five selected measures from the list including:

- Upgrading to more energy-saving appliances;
- Switching to NEVs;
- Upgrading residential energy systems;
- Installing public charging stations in residential areas;
- Adding green facilities in residential areas.

These five measures are selected for several reasons. First, these measures entail certain trade-offs for local city residents. That is to say residents may need to “pay” for the implementation of that measure. Second, the implementation of these measures may involve residents in the decision-making process. Third, residents are the direct beneficiaries and potential maintainer of the associated facilities. For low-carbon measures that do not directly require residents to invest for realizing, e.g., creating carbon accounts and realizing carbon trades, we surveyed respondents’ awareness of those measures.

In addition to estimating WTP amounts for the representative low-carbon measures, this research will test the following hypotheses drawing on existing literature:

**H1.** Residents, who are young, educated, female, and high-income, are more likely to pay for low-carbon measures and pay higher amounts.

**H2.** Residents who bear stronger social and place attachments, measured by family compositions and capitals (e.g., marital status, children, homeownership, and vehicle ownership), are more inclined to pay for low-carbon measures and pay higher amounts.

**H3.** Residents aware of low-carbon measures and policies are more willing to pay for such initiatives and pay higher amounts.

**H4.** Residents are more willing to pay for low-carbon measures and pay higher amounts when provided with additional information on the environmental benefits of those measures.

**H5.** Residents living in residential areas with better built-environment conditions are more willing to pay for low-carbon measures and pay higher amounts.

### Survey design and distribution

To estimate the WTP for different low-carbon measures and test the hypotheses listed in Section 3.1, we designed the questionnaire structured into four sections.

1) The first section surveys respondents’ basic social, economic, and demographic status including age, gender, education, income, employment, marital and family status, housing, and vehicle ownership.
2) The second section assesses residents’ subjective perceptions of climate change and low-carbon development and their trust in city government.
3) The third section surveys residents’ WTP for different low-carbon measures under hypothetical situations. For each low-carbon measure, we first asked respondents’ WTP for that measure, surveyed the reasons for their willingness and unwillingness to pay for that measure, and then asked their WTP again providing them with salient information on the environmental benefits of that measure, e.g., “*this measure can lead to xx carbon emission reduction, equivalent to planting xx trees*”.
4) The fourth section surveys residents’ awareness and knowledge of low-carbon policies implemented in Shenzhen. We first used an open-ended question to let respondents write down any low-carbon policies they have learned about. Then, we used close-ended questions to test respondents’ knowledge of six specific low-carbon policies, five of which are now in effect in Shenzhen and one is not. We started the close-ended questions by providing a brief description of the low-carbon policy and then asked if respondents knew whether this policy was implemented in Shenzhen or not. Respondents should select among 1) “*Yes, I know this policy is implemented in Shenzhen now”*; 2) “*No, I know this policy is NOT implemented in Shenzhen now*”; and 3) “*I don’t know if the policy is currently in effect in Shenzhen or not*” following the method adopted in [39].

The survey takes 20-25 minutes to complete. Respondents were recruited from 14 residential areas across Shenzhen. Those neighborhoods were selected through the following procedures. We first calculated the number of surveys distributed to the different districts in Shenzhen to be proportional to the population size of that district (Table 2). Dapeng New District, Yantian, and Pingshan District were combined for the survey as they are new districts and have a much smaller population compared to other districts. Then, within each district, we randomly selected one to three neighborhoods for survey distribution. We also intentionally include a few “special neighborhoods” in the survey, including an “urban village” in Bao’an District, two near-zero carbon communities, and high-tech talent parks. This selection is to ensure the samples include sufficiently diverse respondents.

**Table 2:** Number of surveys distributed across Shenzhen.

District	Population	# of questionnaire (planned)	# of neighborhoods	# of questionnaire collected
Futian	1,553,225	45	1	45
Luohu	1,143,801	33	1	40
Nanshan	1,795,826	51	2	54
Bao’an	4,476,554	128	3	137
Longgang	3,979,037	113	3	117
Longhua	2,528,872	72	2	72
Guangming	1,085,289	31	1	32
Dapeng New District, Yantian, and Pingshan	921,794	27	1	27
	Total	500	14	524

The total number of surveys is determined with Equation (1) [14,23]:


$$\begin{array}{c} n=\frac{N}{\left(1+Ne^{2}\right)}\#\left(1\right) \end{array}$$

Where *n* is the sample size. *N* is the population size in the area for surveying, and *e* is the designed margin of error. According to the 7th census data, there are more than 17 million people living in Shenzhen in 2020. This equation returns n=399 with a margin of error of 5%. Therefore, we set the total number of surveys for distribution as 500 considering that we may have to remove incomplete surveys. Table 2 shows the number of surveys we have planned and actually received from the different districts.

We hired a professional survey company to distribute surveys. Before the survey distribution, we organized a training session to state the research purpose, survey method, and review each question in the questionnaire with the survey staff. For example, we required staff to not recruit multiple respondents from the same household as family members may share certain beliefs and awareness of climate change and low-carbon development, which may violate the independent and identically distributed (i.i.d) assumption of the statistical analysis.

During the survey process, respondents were firstly presented with an informed consent form indicating voluntary participation and ensuring anonymity of their responses. They then complete the survey under the supervision of staff. Respondents can ask staff for clarifying questions when they find it difficult to understand survey questions. This was particularly helpful for elderly or less-educated participants unfamiliar with technical terms such as *carbon trade* and *carbon credit account*. After completing the survey, staff reviewed responses for completeness. Respondents will receive gifts equivalent to 20 RMB as a reward for their participation. World Resources Institute’s Human Subjects Protection Program reviewed and approved this project, and the need to obtain written consent for the collection, analysis and publication of the obtained and anonymized data for this non-interventional project was waived.

The data were collected between Dec. 30, 2023, and Jan. 20, 2024. Prior to the main data collection, a pre-test was conducted in “Langxin Village” to gather respondents’ feedback, allowing us to refine the clarity of certain survey questions based on the feedback. As we have distributed the questionnaire across 14 different residential areas in Shenzhen, we also collected data describing neighborhood characteristics, such as building age, building type, and the accessibility to green spaces, which will be included in the data analysis. A Cronbach’s alpha coefficient of 0.856 suggests good internal consistency and reliability among collected responses used for subsequent analysis and modeling [48].

### 3.3. Estimating WTP with Contingent Valuation Method (CVM)

We chose the payment scale (PS) to elicit the WTP values for low-carbon measures. Compared to other elicitation approaches, open-ended questions are challenging for respondents as they do not provide any cues on the possible amounts in the question. Close-ended questions do not fully utilize individual responses and require two conjectures to estimate subject responses. Bidding or bargaining is less time-efficient as it involves trained surveyors continuously making offers to respondents. Previous studies have shown that PS and multi-bounded DC can yield more conservative and reliable estimated results for WTP [49,50]. For our research purpose, a conservative estimation is preferred over a progressive one. Therefore, the PS was chosen for this study. The WTP value is calculated as the weighted average of WTP at different bid intervals [51]:


$$\begin{array}{c} WTP_{mean}=\overset{n}{\underset{i=1}{\sum }}f_{i}\alpha_{iL}\#\left(2\right) \end{array}$$

Where $WTP_{mean}$ is the mean WTP value, $f_{i}$ refers to the frequency of WTP at bid interval *i*, and $\alpha_{iL}$ refers to the lower bound of bin *i*.

## Analysis results

The analyses include two parts. In the first part, we performed descriptive analysis to illustrate respondents’ WTP for the five different types of low-carbon measures across scenarios and examined reasons contributing to the willingness and unwillingness to pay. In the second part, we constructed statistical models to explain respondents’ WTP and WTP amounts on low-carbon measures with a set of explanatory factors. The following subsections present the analysis results.

### Descriptive analysis of the survey results

This subsection provides an overview of respondents’ answers to the survey questions.

### Respondents’ social, economic, and demographic structure

The survey includes respondents from diverse social, economic, and demographic groups. Table 3 presents the distribution of respondents’ social, economic, and demographic characteristics. The survey respondents exhibit a slightly more balanced gender ratio (i.e., 1.121) compared to the overall population of 1.227 in Shenzhen. The median age of survey respondents is 36.0, higher than the citywide median age of 32.5. However, this difference can be attributed to our inclusion criteria, which only considers respondents aged 18 years and older. The sample is also more educated compared to the census data, partly due to the inclusion of individuals aged 18 years and below in the census data, many of whom are still pursuing their education. The median income group falls within the range of CNY8,001 to CNY10,000, slightly surpassing the city government’s reported median monthly income of CNY7,234.5 [52]. Overall, the respondents are representative of the Shenzhen population.

**Table 3:** Social, economic, and demographic distribution of survey respondents.s.

Variable	Survey	Census (2020)
Gender	Male: 277 (52.9%) Female: 247 (47.1%)	Male: 55.04% Female: 44.96%
Age Group	18 – 29: 147 (28.1%) 30 – 39: 171 (32.6%) 40 – 49: 107 (20.4%) 50 – 59: 63 (12.0%) >=60: 36 (6.9%)	0 – 14: 15.11% 15 – 59: 79.53% >=60: 5.36%
Education Level	Junior high school or below: 58 (11.1%) High school or equivalent: 112 (21.4%) College (associate’s) degree: 155 (29.6%) Bachelor’s degree: 179 (34.2%) Postgraduate or higher: 20 (3.8%)	Elementary school (12.47%) Junior high school (33.83%) High school or equivalent (22.43%) Bachelor, associate degree, or higher (31.26%)
Occupation Type	Self-employed or small business owner: 63 (12.0%)	–
	Corporate sector (state-owned or private): 267 (51.0%)	
	Public institution (science, education, healthcare, etc.): 22 (4.2%)	
	Unemployed (including unemployed, retired, etc.): 99 (18.9%)	
	Student: 20 (3.8%)	
	Government, military: 5 (1.0%)	
	Freelancers and others: 48 (9.2%)	
Income Level (measured in CNY)	3,000 and below: 63 (12.0%) 3,001 – 4,500: 14 (2.7%) 4,501 – 6,000: 70 (13.4%) 6,001 – 8,000: 92 (17.6%) 8,001 – 10,000: 94 (17.9%) 1,0001 – 15,000: 90 (17.2%) 15,001 – 20,000: 70 (13.4%) 20,001 – 30,000: 16 (3.1%) 30,001 and above: 15 (2.9%)	–
Marital status	Single (including divorced and widow): 177 (33.8%) Married: 347 (66.2%)	–
Parental status	Yes: 336 (64.1%) No: 188 (35.9%)	–
Vehicle ownership	NEV: 87 (16.6%) Gasoline: 287 (54.8%) No vehicle: 150 (28.6%)	–
House ownership	Yes: 250 (47.7%) No: 274 (52.3%)	–

### Respondents’ knowledge and awareness of low-carbon development

**Fig 1:**
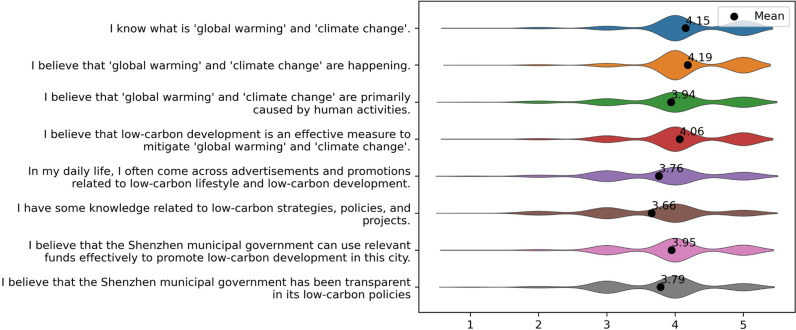
Violin plots showing distributions of respondents’ perceptions of climate change and low-carbon development, and the trust in city government.

The survey includes questions measuring respondents’ subjective perceptions of climate change and low-carbon development, as well as their trust in the city government. Specifically, respondents were presented with eight statements and asked to self-grade their level of agreement using a five-point Likert-type scale, ranging from 1 (indicating “strongly disagree”) to 5 (suggesting “strongly agree”). The eight statements and distribution of responses are summarized in Fig 1.

According to Fig 1, the majority of respondents reported being aware of global warming and climate change (*M* =  4.15, *SD* =  0.75). They also indicated agreement that global warming and climate change are occurring (*M* =  4.19, *SD* =  0.75), are primarily caused by human activities (*M* =  3.94, *SD* =  0.86), and that low-carbon development is an effective measure to mitigate the trend of global warming and climate change (*M = * 4.06, SD =  0.75). Comparatively, respondents expressed less confidence in their knowledge of low-carbon-related measures, policies, and projects (*M* =  3.66, *SD* =  0.87). They also indicated a moderate level of exposure to low-carbon-related advertisements in their daily lives (*M* =  3.76, *SD* =  0.88). Regarding trust in the government, respondents believed that the government could reasonably use funds to promote low-carbon development in Shenzhen (*M* =  3.95, *SD* =  0.77) and perceived the government as transparent in its low-carbon policies (*M* =  3.79, *SD* =  0.79).

**Fig 2:**
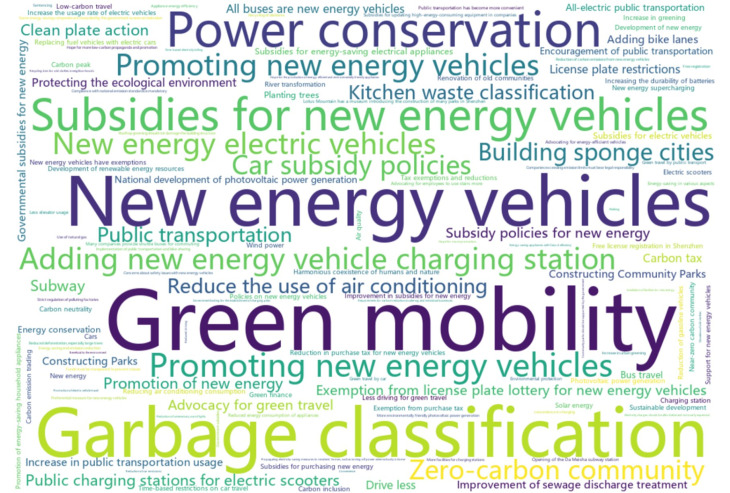
The word cloud for the responses (translated in English) of the open question querying awareness of low-carbon measures in effect in Shenzhen.

In addition to the subjective self-evaluations, we assessed respondents’ awareness of low-carbon measures and policies using open- and close-ended questions, following the method adopted in [39]. Respondents were first presented with a blank box and asked to write down any low-carbon policies or measures they were aware of and currently implemented in Shenzhen. A total of 314 respondents chose to skip this question, indicating that they were not aware of any low-carbon measures in effect. Fig 2 displays the word cloud generated from responses (translated in English) collected from the remaining 210 participants, with larger font sizes suggesting those phrases were mentioned by more respondents. The word cloud reveals that green/low-carbon mobility, NEV-related policies, and garbage classification were among the most frequently mentioned measures. A few respondents also mentioned technical terms such as sponge city, zero-carbon communities, and carbon trading.

**Fig 3:**
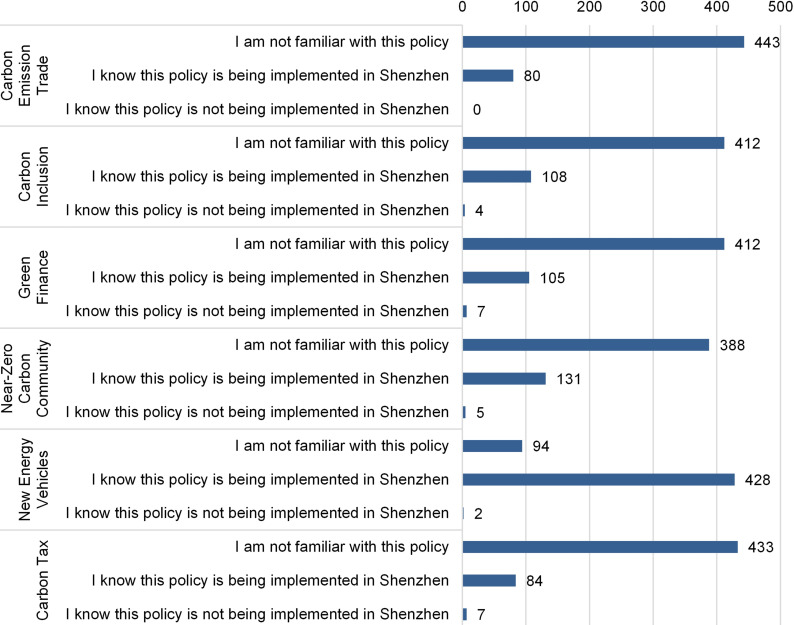
Respondents’ awareness of the implementation of low-carbon policies in Shenzhen.

Following this open-ended question, we used six close-ended questions to gauge respondents’ awareness of six low-carbon policies, provided a brief introduction to the policy, followed by an inquiry about whether respondents were aware of its current implementation status in Shenzhen. The distribution of responses for the six policies is illustrated in Fig 3. It reveals that most respondents are unfamiliar with the listed policies, except for the combined set of policies issued for NEVs. Among the other five policies, respondents demonstrated the highest awareness of the “Near-Zero Carbon Community” (131 votes), followed by “Carbon Inclusion” (108 votes) and “Green Finance” (105 votes). Despite being widely discussed, the “Carbon Tax” is not yet implemented in Shenzhen. Nonetheless, 84 respondents mistakenly believed it was already in effect.

**Fig 4:**
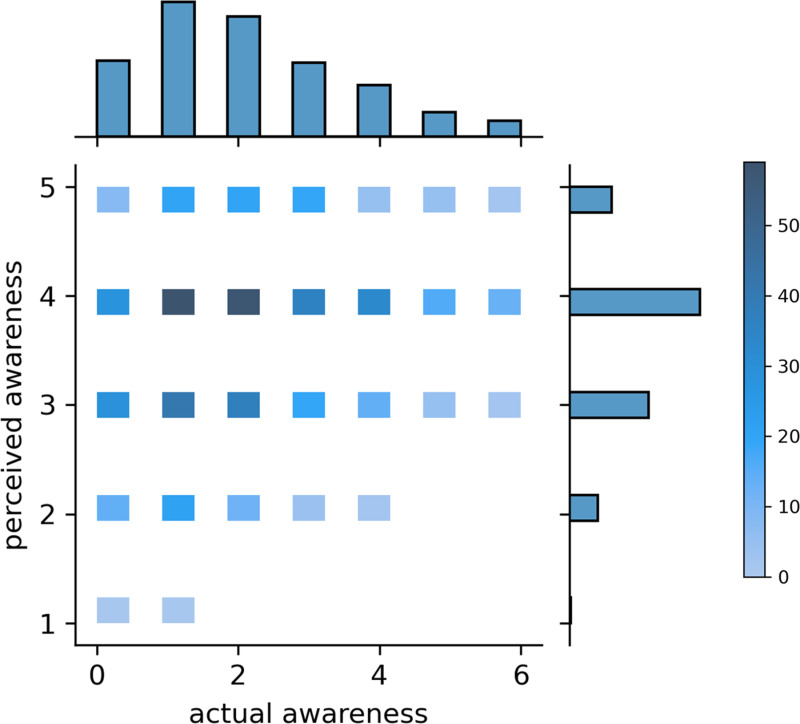
The distribution of respondents’ perceived and actual awareness of low-carbon measures.

We classify respondents’ self-evaluated awareness of low-carbon developments as “*perceived awareness*”. Additionally, we assess respondents’ “*actual awareness*” based on their completion and correctness of the seven open- and close-ended questions, which range from 0 to 6 as no respondent answered all seven questions correctly. Fig 4 displays the distribution of respondents’ *perceived* and *actual* awareness, with darker colors suggesting higher frequencies. According to Fig 4, while most respondents perceive themselves as well-informed about low-carbon policies and measures, they can only correctly answer one to two questions about these measures and policies implemented in Shenzhen. The Spearman’s correlation revealed a weak but significant positive correlation between respondents’ perceived and actual awareness ($r\left(522\right)=.206,p<.001$).

### Respondents’ willingness to pay for differnet low-carbon measures

We surveyed respondents’ WTP for five representative low-carbon measures. For each measure, we provided two scenarios, i.e., with and without additional information on the carbon mitigation effect of that measure. Table 4 presents the ratio and amounts of respondents’ WTP for the different low-carbon measures. The WTP results for each of the five measures are discussed in the following subsections.

**Table 4:** Respondents’ WTP to low-carbon measures with/without information on environmental benefits.

Measurement	WTP ratio	Average WTP amount
Upgrading Level 3 washing machine to Level 2 washing machine.	89.50%	CNY 296.48
Upgrading Level 2 washing machine to Level 1 washing machine.	88.93%	CNY 348.92
Switching to a NEV (without information)	58.60%	CNY 31,987.00
Switching to a NEV (with information)	62.98%	CNY 36,030.3
Upgrading residential energy system (without information)	58.80%	CNY 622.40
Upgrading residential energy system (with information)	64.31%	CNY 654.20
Installing public charging stations (without information)	49.60%	CNY 481.70
Installing public charging stations (with information)	56.30%	CNY 555.90
Adding green facilities (without information)	52.48%	CNY 490.00
Adding green facilities (with information)	57.63%	CNY 539.2

#### WTP on energy-saving appliances.

**Fig 5:**
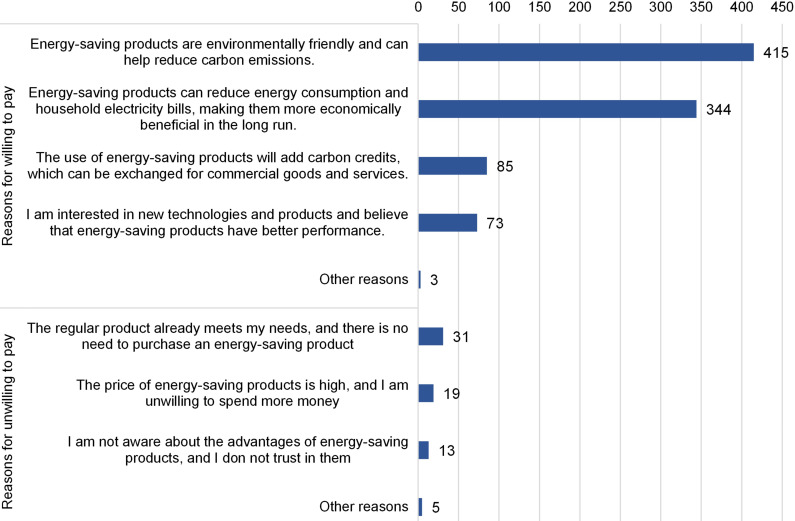
Distribution of reasons for respondents willing and unwilling to upgrade to more energy-saving appliances.

We surveyed respondents’ WTP for energy-saving household appliances, using washing machines as the example to make the questioned scenario more realistic. China employs a tiered rating system, known as the Energy Efficiency Index (EEI), to classify energy-consuming products based on their energy efficiency. The EEI consists of five levels ranking from Level 1 to Level 5, with Level 1 being the most energy-efficient and Level 5 being the least energy-efficient. In the survey, we first asked respondents’ WTP to upgrade to a Level 2 washing machine given that the price of a Level 3 washing machine is CNY 1,000.00. Respondents were presented with two EEI labels for the two washing machines to choose from. Results showed that 89.5% (469 out of 524) of respondents opted to pay for the more energy-efficient Level 2 washing machine, with an average WTP of CNY 1,296.48. The primary reasons cited by respondents for their willingness to invest in more energy-saving appliances included long-term economic benefits, i.e., the reduced utility costs, and the perceived carbon mitigation effects associated with energy-saving household appliances (Fig 5).

Among the 55 respondents who were not willing to upgrade to more energy-saving appliances, 31 stated that regular products already met their needs, while 19 cited the expense of energy-saving appliances as a deterrent. Additionally, 13 respondents mentioned either a lack of awareness or trust in the advantages of energy-saving products (Fig 5). Some respondents are skeptical toward the EEI grading system and question its reliability: “*EEI is something created by manufacturers and there is no accurate reference regarding if they are truly energy-efficient or not*”. Another respondent also expressed that “*the difference [between the Level 2 and Level 3 EEI products] is not significant*”, indicating their distrust in the EEI grading system.

Following this question, we further investigated respondents’ willingness to pay additional money to upgrade to a Level 1 appliance. Respondents were given the choice between EEI Level 2 and Level 1 washing machines. Of these, 466 (88.93%) respondents opted to spend an additional CNY 348.92 for the Level 1 product. Some respondents expressed a preference for top-tier performance, stating “*It’s even okay if the price is CNY 1000 higher than the Level 2 product*” and “*There is no need for the intermediate level*”. However, some respondents viewed the Level 1 product as a form of “*idiot tax*” and preferred products with intermediate EEI levels.

#### WTP on new energy vehicles.

**Fig 6:**
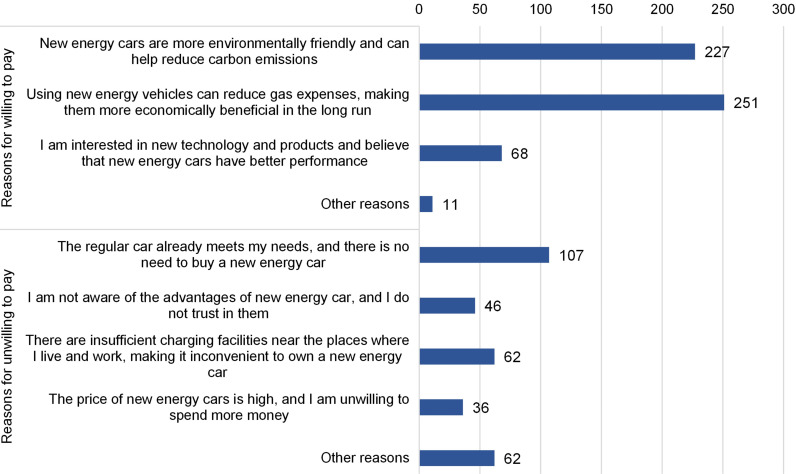
Distribution of reasons for respondents willing and unwilling to pay for NEVs.

We presented respondents with the option to choose between a gasoline vehicle priced at CNY 100,000 and paying an additional amount for an equally functional NEV, without considering the restrictions of plate lottery and governmental subsidies. The results showed that slightly more than half (56.8%) of respondents were willing to switch to NEVs, with an average additional cost of CNY31,987.00. The main reasons cited by respondents for considering NEVs were the associated economic benefits, particularly reduced gasoline costs, followed by the environmental benefits of reducing carbon emissions and energy consumption. Other factors influencing respondents’ decisions included the quick acceleration of some EV brands and their support for the NEV industry (Fig 6). The afterward interviewing of respondents expressed the contributive role of governmental incentive policies, especially the exemption from plate lottery, in promoting the market penetration of NEVs.

Among respondents unwilling to pay for NEVs, 107 considered that gasoline vehicles already met their needs, while 62 rejected NEVs due to insufficient charging facilities near their workplaces and residences. Fewer respondents cited high NEV prices (36 voting) and a lack of knowledge and trust in the advantages of NEVs (46 voting) (Fig 6).

**Fig 7:**
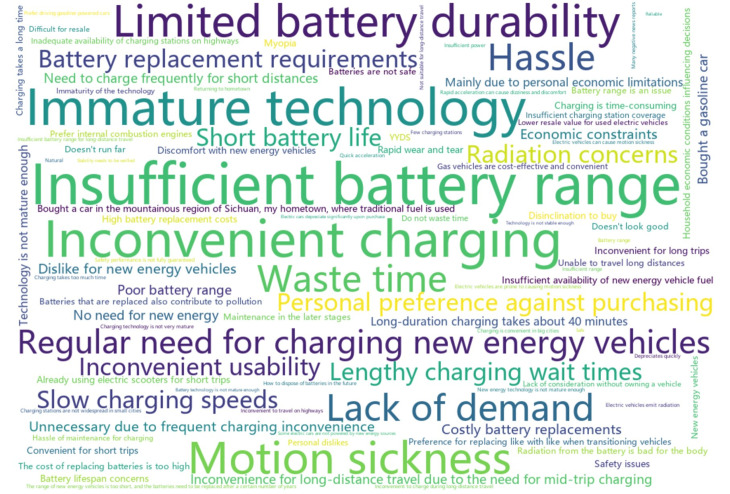
The word cloud for reason, translated in English, specified in the “*Other reason, please specify*” option for respondents unwilling to pay for NEVs.

Upon further investigation into the high occurrence of the “*Other reason, please specify*” option, we created a word cloud (Fig 7) to display the reasons specified by respondents. It shows that respondents were primarily concerned about the limited battery range (17 mentions), insufficient charging infrastructure (18 mentions), short battery lifespan (8 mentions), and potential battery safety issues (10 mentions) of NEVs. The limited battery range and insufficient charging infrastructure in highway service regions and other cities have restricted NEVs from long-distance travel, constituting a major reason for respondents’ reluctance to purchase NEVs. Additionally, seven respondents expressed dissatisfaction with the long charging time of NEVs, making long-distance travel even more inconvenient. Several respondents also expressed concerns regarding battery lifespan and the associated costs of repairing or replacing aging batteries. Two respondents highlighted the environmental pollution caused by battery disposal, undermining the eco-friendliness of NEVs. Furthermore, ten respondents raised safety concerns regarding NEV batteries. They consider the current technology is still immature. Three respondents mentioned experiencing motion sickness in NEVs, attributing it to radiation emitted by NEV batteries. Another respondent, however, attributed motion sickness to the rapid acceleration of NEVs. In addition to these concerns, several respondents expressed a preference for the driving experience of gasoline vehicles over NEVs. Economic considerations, such as the lower resale value of NEVs compared to gasoline vehicles, were also cited by a few respondents.

We used an additional question to survey respondents’ WTP for NEVs when provided with extra information on their carbon mitigation effect. The results indicated a slight increase in the ratio of respondents’ willing to pay for NEVs, rising from 58.6% to 62.98%. Additionally, 31.6% (97 out of 307) of respondents increased their payments when presented with additional information, yielding an increment of CNY 4,043.3 in the averaging WTP amount (Table 4).

#### WTP on upgrading residential energy system.

The Shenzhen government has promoted technologies aimed at improving the energy efficiency of residential buildings, such as photovoltaic building facades and centralized cooling systems. We designed questions to survey respondents’ WTP for upgrading the energy systems of residential buildings. The results revealed that only 58.8% of respondents were willing to pay for this upgrade with an average amount of CNY 622.40. The primary reasons cited by respondents for supporting the upgrading of building energy systems were the economic benefits associated with potentially reducing electricity costs, followed by the environmental benefits of carbon mitigation. Additionally, 121 respondents considered this measure could improve their living conditions.

**Fig 8:**
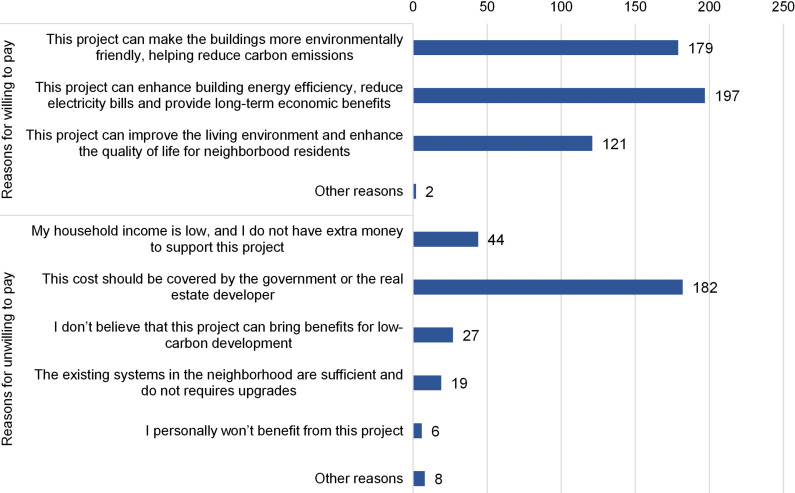
Distribution of reasons for respondents willing and unwilling to pay for upgrading residential energy system.

Respondents unwilling to pay for upgrading building energy systems mostly believed that the cost should be covered by the government or the real estate developer (Fig 8). Forty-four respondents rejected upgrading the residential energy system due to low household income and 27 respondents doubted the benefits of the project for low-carbon development. Nineteen respondents consider the existing energy system to be sufficient and not in need of upgrades. One respondent expressed reluctance toward any civil projects implemented in residential areas, stating “*It’s always repairs and construction, which generates noises and debris impacting daily lives*”. Relatively few refuters indicated that they were unlikely to benefit from this upgrading project.

The provision of additional information on the carbon mitigation effect of this project slightly increased respondents’ WTP from 58.80% to 64.31%, as well as an additional CNY 31.8 on the WTP amounts (Table 4).

#### WTP on installing public charging facilities in residential areas.

**Fig 9:**
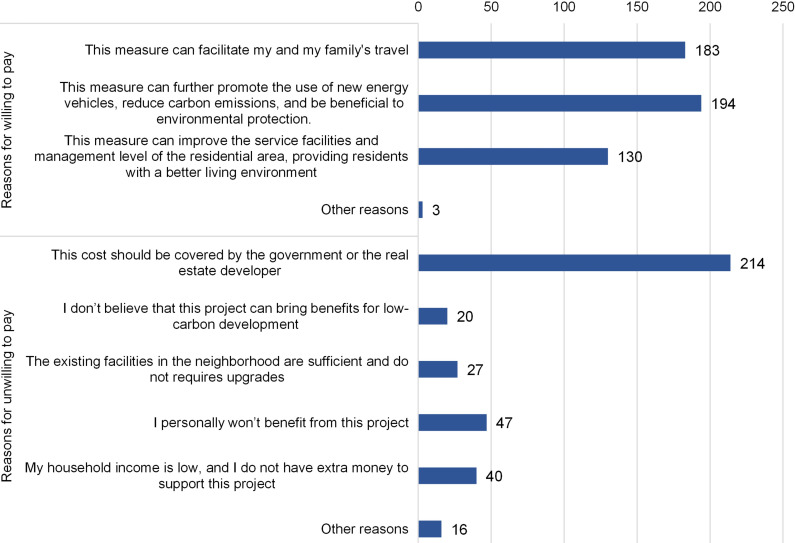
Distribution of reasons for respondents willing and unwilling to pay for installing public charging stations.

To promote NEVs, the Shenzhen government has initiated efforts to install EV charging stations in residential areas. We designed questions to survey respondents’ WTP for installing public EV charging stations. Results indicated that only 59.4% of respondents, totaling 260 individuals, were willing to support this project, with an average WTP of CNY 481.7. However, when provided with additional information on the environmental benefits of the project, the ratio of respondents’ WTP increased to 56.30%, with an average increment of CNY 74.2 in the WTP amount (Fig 9).

Among respondents willing to pay for installing public charging stations, 194 believed that this initiative could further promote the adoption of NEVs and reduce carbon emissions, thus benefiting the environment. Additionally, 183 respondents saw this measure as facilitating their travel, while 130 considered it improving service facilities and management in the neighborhood, thereby enhancing the overall living environment (Fig 9).

For respondents unwilling to contribute to the public charging stations, the majority (211 out of 264) believed that the funding should be provided by the government or real estate developers. Forty-seven respondents felt they would not benefit from the project, while 40 cited low household income as a barrier to their contribution. Additionally, 20 respondents doubted the contribution of this measure to low-carbon development. Three respondents expressed concerns about sacrificing public spaces for installing charging stations due to limited neighborhood space, while another three suggested integrating installation and management fees into charging fees. Interestingly, a few respondents without EVs expressed willingness to pay for public charging stations to accommodate their visitors.

#### 4.1.3.5. WTP on adding green facilities in residential areas.

**Fig 10:**
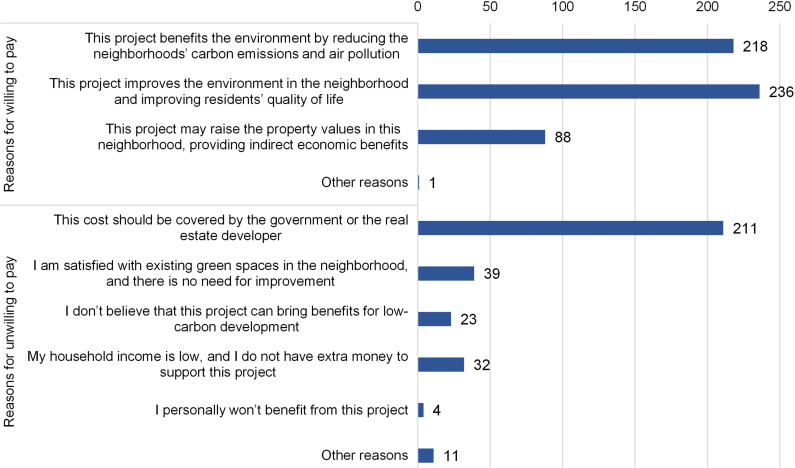
Distribution of reasons for respondents willing and unwilling to pay for adding green facilities in residential areas.

Installing carbon sinks in residential areas is an effective measure for low-carbon development. We surveyed respondents’ WTP for adding green facilities within residential areas, such as community parks and rooftop gardens. 52.48% or 275 respondents expressed support for this project with an average payment of CNY 490.00. The provision of additional information on the carbon mitigation effect can increase this ratio to 57.63% and average payment to CNY 539.2 (Fig 10).

Among respondents willing to pay for adding green facilities, the majority (236) believed that this measure could improve living conditions, followed by 218 respondents who recognized the environmental benefits, such as reducing carbon emissions and air pollution. Eighty-eight respondents also considered that this project could increase housing prices and provide indirect economic benefits (Fig 10).

For respondents unwilling to pay for the project, the majority (211) believed that the funding should be provided by the government or real estate developers. Thirty-nine respondents felt that the existing neighborhood greenery was sufficient and were reluctant to make improvements. Thirty-two respondents cited low household income as a barrier to their contribution, while 23 were skeptical about the project’s effectiveness in low-carbon development. Relatively few (4) respondents felt they would not benefit from the project. One respondent has particularly noted that increasing green spaces may lead to an increase in mosquitoes, and hence reluctant to add green facilities within the residential areas.

### Statistical analysis of survey results for hypothesis testing

We conducted a set of statistical analyses and constructed models to test the hypotheses outlined in the Method section. The following subsections present the analysis results.

### Factors influencing respondents’ WTP for low-carbon measures

We developed statistical models to analyze respondents’ WTP for various low-carbon measures. In addition to personal-level variables capturing respondents’ social, economic, and demographic characteristics, we also considered built-environment conditions for each of the 14 survey neighborhoods, including built year, floor area ratio, greening rate, and ratio of nearby green land. Given the varied variables considered, we performed the multicollinearity test before the model construction. It was found that variables “*hasKids*” and “*maritalStatus*” are highly correlated ($r\left(522\right)=.888,p<.001$). We therefore removed “*hasKids*” from the model construction. After removing the variable, the Variance Inflation Factor (VIF) values for other variables generally range from 1.0 to 2.2, suggesting a low risk of multicollinearity in the model.

We chose stepwise binomial logistic regression to model respondents’ binary responses regarding whether pay or not pay for different low-carbon measures. The model used a stepped approach to iteratively select variables, the inclusion of which can significantly improve the predictive performance of the model. In this way, the model itself works as a feature selector and identifies influential factors. We set the threshold of significance level for a variable to enter to model to be 0.25, to stay in the model to be 0.15. We used SAS Studio, a professional statistical software, for the modeling. Table 5 shows the modeling results. All models were found to be statistically significant with p-values <  0.0001 for the corresponding chi-square tests.

**Table 5:** Binomial logistic regression models for respondents’ WTP on low-carbon measures.

Variable	Upgrading to energy-saving appliances	Switching to NEVs	Upgrading residential energy systems	Installing public charging stations	Adding green facilities
Personal level variables					
age	– (-)	– (-)	– (-)	– (-)	– (-)
gender	– (-)	– (-)	– (-)	– (-)	– (-)
houseOwnership	– (-)	– (-)	– (-)	– (-)	– (-)
5YearResidency	– (-)	– (-)	– (-)	– (-)	-0.3978 (0.1266)
educationLevel	– (-)	– (-)	0.2767^***^ (0.0017)	– (-)	– (-)
incomeLevel	0.2700^***^ (0.0027)	– (-)	– (-)	– (-)	– (-)
maritalStatus	0.6455^**^ (0.0285)	– (-)	– (-)	-0.2943 (0.1232)	– (-)
vehicleOwnership_NEV	– (-)	0.8117^***^ (0.0001)	– (-)	– (-)	– (-)
vehicleOwnership_ gasoline	– (-)	-0.7871^***^ (<.0001)	– (-)	– (-)	– (-)
ocupationType_ self-employed or small business owner	– (-)	– (-)	– (-)	– (-)	– (-)
ocupationType _ public institution	– (-)	– (-)	– (-)	– (-)	– (-)
ocupationType _corporates	– (-)	– (-)	– (-)	– (-)	– (-)
ocupationType _student	– (-)	– (-)	– (-)	– (-)	– (-)
ocupationType _govenment, military	– (-)	– (-)	– (-)	– (-)	– (-)
ocupationType_freelancer and other	– (-)	– (-)	– (-)	– (-)	– (-)
lowCarbonAwareness	0.3351^***^ (0.0037)	0.2725^***^ (<.0001)	0.2706^***^ (<.0001)	0.2613^***^ (<.0001)	0.3338^***^ (<.0001)
Neighborhood level variables					
floorAreaRatio	– (-)	0.0950 * (0.0666)	– (-)	– (-)	– (-)
builtYear	– (-)	– (-)	– (-)	– (-)	– (-)
greeningRate	– (-)	2.7417 (0.1432)	– (-)	4.4140^**^ (0.0130)	– (-)
ratioGreenLandinBuff	– (-)	– (-)	– (-)	– (-)	– (-)
Model Goodness of Fitness					
R-square	0.0520	0.1181	0.0676	0.0599	0.0607
Adjusted R-square	0.1062	0.1590	0.0910	0.0798	0.0810
Chi-square statistic	23.5345	53.0240	32.9635	29.7518	29.8069
Prob (Chi-square statistic)	<.0001	<.0001	<.0001	<.0001	<.0001

Note: *  *p* <  0.1; ** *p* <  0.05; *** *p* <  0.01.

According to Table 5, respondents’ actual awareness of low-carbon measures, which is measured by their completion and correctness of answers to the seven open- and close-ended questions related to low-carbon measures/policies in effect in Shenzhen, emerge as the strongest predictor of respondents’ WTP for all five low-carbon measures. Additionally, married and high-income respondents are more likely to purchase energy-saving appliances. Respondents with higher levels of education tend to pay for upgrading the energy system in their residential buildings. Respondents who had moved to Shenzhen within the past five years are more likely to support the installation of green facilities within neighborhood areas. Interestingly, respondents owning NEVs exhibit a significantly higher probability of being willing to pay for NEVs, whereas those owning gasoline vehicles are notably less likely to pay for NEVs compared to non-vehicle owners. This suggests that owners of both vehicle types are content with their respective choices of vehicle types.

Regarding built-environment factors, respondents living in neighborhoods with higher floor area ratios and greening rates - characterized by tall buildings with ample vegetation - are more willing to pay for NEVs. Additionally, respondents living in neighborhoods with higher greening rates are more inclined to pay for the installation of public charging stations. During the survey, some respondents expressed that their neighborhoods lack sufficient open spaces for the installation of additional facilities, which is also reflected in the modeling results.

In addition to the binary responses indicating willingness or unwillingness to pay for the different low-carbon measures, we also constructed interval regression models to examine factors influencing payment amounts for these measures. Specifically, for respondents selecting the *t*th level of payment, denoted as $x_{t}$, we consider their actual payment amount should fall within the range of $\left(x_{t-1}+1,x_{t}\right]$, which is interval-censored and makes interval regression a natural fit for this analysis. The modeling results are presented in Table 6.

**Table 6:** Interval regression models for respondents’ payment amounts on low-carbon measures.

Variable	Upgrading to energy-saving appliances	Switching to NEVs	Upgrading residential energy systems	Installing public charging stations	Adding green facilities
Personal level variables					
age	– (-)	– (-)	–	– (-)	-7.4834^***^ (0.0083)
gender	– (-)	5108.328^**^ (0.0358)	(-)	– (-)	102.8520^ *^ (0.0637)
houseOwnership	– (-)	5691.657^**^ (0.0372)	– (-)	– (-)	– (-)
5YearResidency	- 51.5669^**^ (0.0194)	– (-)	-190.3404^**^ (0.0286)	– (-)	-109.2902 (0.1533)
educationLevel	13.6571 * (0.0909)	– (-)	– (-)	-54.0075 * (0.0559)	-96.4493^***^ (0.0029)
incomeLevel	7.4048 (0.1253)	1539.834 * (0.0834)	73.2065^***^ (<.0001)	75.5706^***^ (0.0001)	112.5593^***^ (<.0001)
marritalStatus	– (-)	– (-)	– (-)	– (-)	– (-)
vehicleOwnership_NEV	78.6555^***^ (0.0009)	5774.565 (0.1011)	– (-)	173.6730^**^ (0.0311)	165.3325 * (0.0508)
vehicleOwnership_ gasoline	25.9460 (0.1466)	– (-)	– (-)	– (-)	110.5344 * (0.0926)
ocupationType_ self-employed or small business owner	– (-)	– (-)	– (-)	– (-)	-259.260^**^ (0.0229)
ocupationType _ public institution	– (-)	-15211.3^**^ (0.0235)	– (-)	– (-)	– (-)
ocupationType _corporate sector	– (-)	– (-)	– (-)	– (-)	-220.259^**^ (0.0206)
ocupationType _student	– (-)	– (-)	– (-)	263.6096 * (0.0517)	– (-)
ocupationType _govenment, military	– (-)	– (-)	– (-)	– (-)	– (-)
ocupationType_freelancer and other	– (-)	– (-)	– (-)	197.8935 * (0.0828)	– (-)
lowCarbonAwareness	8.1523 * (0.0896)	– (-)	– (-)	– (-)	24.6027 (0.1443)
Neighborhood level variables					
floorAreaRatio	– (-)	–	– (-)	38.3602 * (0.0801)	34.1052 (0.1025)
builtYear	11.5089** (0.0308)	1434.379 * (0.0907)	63.0800*** (0.0023)	82.4460^***^ (0.0020)	55.1668** (0.0362)
greeningRate	– (-)	–	– (-)	– (-)	– (-)
ratioGreenLandinBuff	– (-)	(-)	– (-)	– (-)	-483.676^**^ (0.0279)
Model Goodness of Fitness					
AIC	1897.717	1208.947	1672.354	1398.919	1517.806
BIC	1930.801	1256.700	1691.005	1448.769	1586.525
R-square (lower bound)	0.0533	0.0999	0.0759	0.1425	0.1845
R-square (upper bound)	0.0610	0.0995	0.0754	0.1379	0.1805

Note: *  p <  0.1; ** p <  0.05; *** p <  0.01.

According to Table 6, it is not surprising to find that respondents with higher income levels tend to pay higher amounts for all surveyed low-carbon measures, especially for those aimed at collective goods such as building renovation and residential area upgrades. While respondents’ awareness of low-carbon initiatives plays a significant role in binary logistic models (Table 5), its influence on payment amounts is comparatively less pronounced. However, those with heightened awareness tend to pay higher amounts for energy-saving appliances and the installation of green facilities in their neighborhoods. Moreover, respondents who moved to Shenzhen within the past five years tend to contribute higher amounts for three low-carbon measures including upgrading to energy-saving appliances, upgrading residential energy systems, and adding green facilities. This inclination could stem from a heightened sense of civic responsibility among this group towards contributing to the city’s sustainability efforts. The influence of education levels on payment amounts varies across low-carbon measures. While respondents with higher education attainment are more inclined to invest in energy-saving appliances, they show reluctance towards allocating higher funds for public charging stations and green facilities. Subsequent surveys within this demographic reveal that many attribute the responsibility for neighborhood environmental improvements to the government or real estate developers.

Owners of NEVs exhibit a greater willingness to allocate higher amounts towards most low-carbon measures, except for upgrading residential energy systems when compared to those without vehicles or owning conventional gasoline vehicles. Male homeowners display a higher propensity to invest more in NEVs, while respondents employed in public institutions tend to pay lower amounts towards these vehicles. Additionally, students and freelancers show a greater willingness to invest in public charging stations. Young males, students, the unemployed (housewives and the retired), and respondents working for public institutions tend to contribute higher amounts for green facilities within residential areas.

Regarding built-environment factors, respondents residing in aging residential areas show a higher propensity to invest more in all surveyed low-carbon measures. Similarly, those residing in neighborhoods characterized by high floor area ratios, e.g., high-rise residential buildings, are more inclined to pay higher amounts for installing low-carbon facilities within residential areas, potentially indicating a greater demand for such facilities. Conversely, respondents living in areas with abundant access to green spaces tend to allocate fewer funds towards green facilities, likely due to their lower demand for additional ecological services.

### The impact of additional information on residents’ WTP

We surveyed respondents’ WTP and the corresponding amounts for four types of low-carbon measures, excluding upgrading to energy-saving appliances, under scenarios with and without detailed information on carbon mitigation effects. According to Table 4, it is evident that providing additional information can boost both the likelihood and the amounts respondents are willing to pay for these measures. Furthermore, we conducted a two-sample paired t-test to compare respondents’ WTP ratios and payment amounts under the two scenarios (Table 7). The results indicate a significant increase in both the probability of WTP and the amounts respondents are willing to pay when provided with additional information on the carbon mitigation effect.

**Table 7:** Two-sample paired t-test of WTP ratio and payment amount between scenarios with and without known environmental benefits.

	WTP ratio	WTP amount
Switching to NEVs	*t*(523) = -4.90, *p* < 0.001^***^	*t*(329) = -6.86, *p* < 0.001^***^
Upgrading residential energy systems	*t*(523) = -5.54, *p* < 0.001^***^	*t*(336) = -5.87, *p* < 0.001^***^
Installing public charging stations	*t*(523) = -5.64, *p* < 0.001^***^	*t*(289) = -6.61, *p* < 0.001^***^
Adding green facilities	*t*(523) = -5.33, *p* < 0.001^***^	*t*(301) = -6.22, *p* < 0.001^***^

Note: *  *p* <  0.1, ** *p* <  0.05, *** *p* <  0.01.

An interesting aspect is understanding who is more likely to be influenced by the provided information. To explore this, we compared respondents who readily changed their minds or increased their payment amounts for at least two low-carbon measures against those who maintained their initial opinions, considering various socio-demographic characteristics. The analysis reveals that new Shenzhen residents who moved to the city within the past 5 years are more susceptible to being swayed by additional information (*t*(522) =  -1.70, *p* = .090*).

## Discussion

### Summary of results for hypothesis testing

In this research, we performed a combination of analyses to investigate the WTP of Shenzhen residents for five representative low-carbon measures currently implemented in the city, as outlined in Table 4. Additionally, we developed statistical models to test hypotheses explaining the variations in WTP among respondents, which are summarized below.

Previous related studies have indicated that respondents’ social, economic, and demographic characteristics influence their WTP for low-carbon measures as well as the payment amounts (**H1**). Specifically, numerous studies have shown that high-income and educated respondents are more likely to pay higher amounts for low-carbon measures [14,17,25,36,37]. Our analysis yielded more nuanced results. The binary variable indicating willingness versus unwillingness to pay for low-carbon measures did not show significant variation across different income levels. However, among the subset of respondents who indicated their willingness to pay for low-carbon measures, they tended to contribute higher amounts. The impact of education attainment also varied across different low-carbon measures. Respondents with higher education attainments were more likely to pay for upgrading residential energy systems and pay higher amounts for energy-saving appliances. Conversely, they tended to pay lower amounts for initiatives targeting collective benefits, such as installing of public charging stations and green spaces in residential areas. This finding, though contradicting our initial expectations, is supported by research indicating that individuals from lower social classes exhibit greater concern for collective welfare and demonstrate more pro-social behavior [53,54]. During the survey, we found that respondents with lower education levels often exhibited greater generosity when contributing to projects intended to benefit the community. In contrast, individuals with higher education levels tended to view such contributions as investments and carefully assessed the anticipated returns. For instance, one respondent commented that “*the associated environmental benefits are lower than expected and are not worth the cost*”. These educated elites also tended to attribute the responsibilities for low-carbon development to city government, real estate developers, and end consumers using low-carbon facilities. Future studies may consider further exploring such “*educated elite*” effect with approaches distinguishing between personal benefits and collective welfare in eliciting public WTPs (e.g., [55]). Regarding other socio-demographic factors, age groups generally did not significantly influence the probability or extent of financial contributions despite younger respondents were more likely to pay higher amounts for green facilities within residential areas. Moreover, male respondents were willing to pay higher amounts for low-carbon measures, particularly for upgrading to NEVs.

Secondly, existing studies have suggested that residents with stronger social and place attachments, measured by family compositions and capitals (e.g., marital status, children, homeownership, and vehicle ownership), are more inclined to pay for low-carbon measures and contribute higher amounts (**H2**) [17,40,44]. However, our analysis results generally refute this hypothesis. There is no significant correlation between respondents’ WTP and their ownership of properties, marital status, or family compositions, despite the strong preference among NEV owners for purchasing NEVs. Conversely, our analysis revealed that new Shenzhen residents, who relocated to the city within the past five years, are more inclined to contribute higher amounts for low-carbon measures. This may be due to Shenzhen’s reputation as a “migrant city” that attracts new migrants from across the country. Future studies may be needed to explore urban planning or management methods that help new migrants build social and place attachments, e.g., [56,57].

Thirdly, numerous studies based on the *theory of planned behavior* have shown that respondents’ awareness and pre-existing knowledge of low-carbon measures and policies influence their WTPs (**H3**) [14,25,37,41]. Our analysis results suggest that Shenzhen residents were generally not well informed about low-carbon measures in effect. Respondents who demonstrated awareness of these measures, assessed by their accuracy and completeness in answering questions about low-carbon measures, consistently expressed higher WTP for all five types of surveyed low-carbon measures, and were willing to pay higher amounts for certain low-carbon measures. Thus, the analysis results support **H3**.

In conjunction with the insights gained regarding low-carbon awareness, our analysis also underscored the influence of providing residents with detailed information about the environmental benefits associated with those measures (**H4**). Two-sample paired *t-*tests suggest a significant increase in both the probability of WTP and the corresponding payment amounts following the provision of detailed information about the carbon mitigation effects of the surveyed low-carbon measures. This finding aligns with previous studies such as [37,39]. The analysis further indicates that respondents who had relocated to Shenzhen within the past five years are particularly receptive to this additional information.

Lastly, some studies have also indicated the potential impacts of environmental factors on respondents’ WTPs [26,38]. We hypothesized that the built-environment conditions in neighborhoods could influence respondents’ WTP for low-carbon measures (**H5**). Survey findings suggest that residents in areas with higher rates of greenery are more inclined to support the installation of public charging stations, potentially due to the availability of ample space to accommodate such facilities. Furthermore, residents in aging residential areas demonstrate a greater propensity to pay higher amounts for low-carbon measures.

## Implications

The analysis yields several practical implications for promoting low-carbon urban developments.

There is a clear need for educational interventions to enhance public awareness of low-carbon development initiatives. The survey data revealed a general lack of comprehensive understanding among respondents concerning low-carbon measures and policies in effect. Measures related to mobility, such as NEVs and green transportation options, were relatively better known. However, our analysis indicates that increasing awareness levels or providing information about the environmental benefits can significantly boost both the WTP likelihood and corresponding payment amounts for low-carbon measures. Thus, it is advisable for the government to actively promote the economic and environmental advantages associated with low-carbon projects to the general public. This could be achieved through simple and accessible messaging, such as highlighting the number of trees saved or planted equivalent to those low-carbon initiatives. Advertisements should also emphasize both direct and indirect impacts. For example, installing public EV chargers can encourage more residents to switch to EVs and hence reduce the noises caused by combustible engines. Highlighting these indirect and collective benefits of low-carbon projects may invoke higher WTP among residents who do not directly benefit from the low-carbon projects. For example, the authors in [58] listed the four types of non-use values for environmental improvements including *existence value*, *option value*, *altruistic value*, and *bequest value*, which could be considered in the future advertisement and messaging strategies aimed at increasing public engagement with low-carbon initiatives.

In addition to educational intervention, governments may also explore targeted approaches. The findings suggest that new Shenzhen residents exhibit a greater willingness to financially support low-carbon initiatives and are more receptive to informational campaigns. This presents an opportunity for city managers to design initiatives or awareness campaigns specifically tailored to the needs and perspectives of these new residents. Furthermore, for existing residents, neighborhood-level campaigns and community events could be employed to foster a sense of collective responsibility and ownership of their neighborhoods. Governments should consider investing in methods to minimize disturbances, such as noise, debris, and service disruptions, for local residents during the implementation of low-carbon renovation projects. In the survey, several respondents expressed hesitation to financially support low-carbon projects aimed at upgrading their residential or neighborhood areas due to the noise and inconvenience associated with construction work. This underscores the needs for adopting advanced technologies, such as low-noise machinery and dust-suppression construction methods, as well as optimizing workflows to mitigate disturbances caused by renovation projects.

Moreover, we note a significant divergence in attitudes toward NEVs between existing NEV owners and gasoline vehicle owners. Technological concerns, particularly related to batteries, range, lifespan, and charging time, remain substantial barriers for gasoline vehicle owners considering switching to NEVs. However, NEV owners are generally satisfied with their vehicles, suggesting that these technological concerns may be exaggerated and influenced by a lack of updated information. To bridge this information gap, it is recommended that media outlets and NEV manufacturers actively advertise technology advancements. This should include debunking misinformation, such as the misconception that motion sickness is caused by “*battery radiation*”. Furthermore, NEV manufacturers could consider providing free trials to potential customers to increase market penetration, which allow potential buyers to experience the benefits of NEVs firsthand and may help alleviate their technological concerns.

## Limitations

This research has several limitations. We did not include all low-carbon measures and policies in effect in Shenzhen in the survey. This decision was made to prevent survey from becoming overly lengthy and potentially distracting for respondents. Secondly, like other surveys, the data collection relied on self-reported responses from participants, which may introduce response bias or inaccuracies due to factors such as social desirability bias or recall errors. Additionally, the research utilized a cross-sectional design, capturing data over a relatively short period, which may limit the ability to establish causality or observe changes in WTP over time, providing only a snapshot of respondents’ attitudes and behaviors.

## Conclusion

The culmination of this study offers valuable insights into the perceptions and preferences of Shenzhen residents regarding low-carbon initiatives. Through a comprehensive analysis of residents’ WTP for various low-carbon measures, we have illuminated key factors influencing their support and financial contributions towards sustainable development. Our findings underscore the significance of educational interventions in enhancing public awareness of low-carbon strategies, highlight the potential effectiveness of targeted interventions tailored to new and existing residents, and emphasize the importance of advertising new technological advancements in NEVs, alongside subsidies. Future research endeavors could build upon these findings. By addressing these considerations, policymakers and urban planners can refine strategies to foster low-carbon development initiatives not only in Shenzhen but also in other urban settings.
